# Coverage Path Planning and Point-of-Interest Detection Using Autonomous Drone Swarms

**DOI:** 10.3390/s22197551

**Published:** 2022-10-05

**Authors:** Konstantinos Bezas, Georgios Tsoumanis, Constantinos T. Angelis, Konstantinos Oikonomou

**Affiliations:** 1 Department of Informatics and Telecommunications, Campus of Arta, University of Ioannina, 47100 Arta, Greece; 2 Department of Informatics, Ionian University, 49100 Corfu, Greece

**Keywords:** drones, coverage path planning, point-of-interest detection

## Abstract

Unmanned Aerial Vehicles (UAVs) or drones presently are enhanced with miniature sensors that can provide information relative to their environment. As such, they can detect changes in temperature, orientation, altitude, geographical location, electromagnetic fluctuations, lighting conditions, and more. Combining this information properly can help produce advanced environmental awareness; thus, the drone can navigate its environment autonomously. Wireless communications can also aid in the creation of drone swarms that, combined with the proper algorithm, can be coordinated towards area coverage for various missions, such as search and rescue. Coverage Path Planning (CPP) is the field that studies how drones, independently or in swarms, can cover an area of interest efficiently. In the current work, a CPP algorithm is proposed for a swarm of drones to detect points of interest and collect information from them. The algorithm’s effectiveness is evaluated under simulation results. A set of characteristics is defined to describe the coverage radius of each drone, the speed of the swarm, and the coverage path followed by it. The results show that, for larger swarm sizes, the missions require less time while more points of interest can be detected within the area. Two coverage paths are examined here—parallel lines and spiral coverage. The results depict that the parallel lines coverage is more time-efficient since the spiral increases the required time by an average of 5% in all cases for the same number of detected points of interest.

## 1. Introduction

Drones presently are used for a variety of applications. Technological advances, mainly in sensory devices and nanotechnology, provide the tools that enable the development of miniature drones that can last for several minutes to hours per flight or even fly continuously. Historically, drones have been funded by the US military, with the first flights dating to 1903 [1]. During the first demonstrations, drones had very limited features. They were remotely operated by one person and wireless communications were used to transmit basic commands. The most attractive aspect of this technology was the fact that it was unmanned, which meant that no pilot risked his life on the battlefield.

Presently, drones have advanced to the point where no human operator is required to complete a mission. They are being adopted by many industries since they can potentially lower the cost of product delivery and minimize human casualties under states of emergency. Drone technology can be applied to many aspects of human life. A few examples are post-earthquake response for human detection and damage assessment [2], early detection of forest fires [3], wildfire tracking [4], parcel or food delivery systems [5,6], structural integrity [7] and power-line [8] inspection.

Ever since the miniaturization of computer components, sensory devices have become much easier to include in a compact and easy-to-develop system with a basic operation such as the collection of information from the surrounding environment and wireless data transmission. Such systems can be adjusted to operate on drones and even create swarms that can be coordinated to complete tasks as a team. Although the traditional operation of a single drone requires a pilot, this model cannot be employed for drone swarm applications since a typical swarm can include tens of drones, hence the system is prone to failure due to human error.

The swarm collects information in real time and decides the necessary actions towards the completion of a mission. A major issue regarding drone swarms is navigating a natural environment. Algorithmic steps can provide a path planning scheme that can be altered in real time. The path a drone must follow is not predefined but is determined under an algorithm that includes simple steps individually followed by the drones to produce the necessary distributed behavior.

The role of an operator in drone swarms can be either absent or in the form of simple commands [9] which coordinate the entire swarm towards task completion. During the last decade, applications with multiple drones are headed towards full autonomy. Coverage Path Planning (CPP) is the field that describes the algorithms which are employed for full coverage of an Area of Interest (AoI).

Applications in open seas such as search and rescue (SaR) [10], oil-spill monitoring and cleaning using underwater autonomous robotics [11], and real-time oil-spill mapping [12] can also benefit from swarm algorithms and drone swarms in general. SaR missions and oil-spill monitoring or mapping are time-critical applications. In the case of SaR missions, multiple scenarios can be assumed, such as emergency plane landing at seas or boat sinking events. Both cases require the full coverage of large open-sea areas a task well suited for a swarm of areal [12], underwater [11] or even surface autonomous robotics [13] which can sense environmental attributes such as thermal dissipation using special equipment.

Autonomous drone swarms can be applied to scenarios such as forest fire-fighting [14,15] or early forest fire detection [16]. Both applications require a well-defined algorithm to cover the area efficiently and provide low response times. The only initial input given to the swarm is the geographical AoI. Please note that the swarm can operate with minimal to no intervention by an operator.

The main contribution of the current work is the development of a CPP algorithm that can be employed for either a single drone or a swarm of drones. The algorithm can achieve full area coverage and Point-of-Interest (PoI) detection with no human intervention. Two models are developed for PoI detection, the basic and the advanced. In the first case when a drone detects several PoIs within its coverage radius it chooses its closest one. The advanced model takes into consideration the amount of time each PoI remains within the drone’s coverage radius and chooses the one with the smallest time. As such, there is increased chance that the swarm might collect information from all PoIs it detects.

Two paths are examined, the parallel and the spiral. Comparison is conducted between them to determine which is the most suitable for a CPP that also takes into consideration the data collection process and the characteristics of the current algorithm.

The main strengths of the current algorithm ate the following: (i) The swarm employs distributed actions which require information exchange between the drones during a CPP mission, as such no human intervention is required to coordinate the swarm for full area coverage; (ii) The algorithm allows for adaptive speed during a CPP mission based on the current requirements which helps improve the PoI detection and limit data loss; and (iii) The drones acquire linear formation with exact distances between them and avoid overlapping coverage.

The weaknesses of the current algorithm are the following: (i) Obstacles and physical collision avoidance as well as path rescheduling are not handled by the algorithm which limits the potential applications that it can be used for; and (ii) The algorithm assumes a full graph topology between the swarm which also limits the applications it can be used for which must have an environment with line-of-sight communications.

The simulated results showcase that different swarm sizes can be employed and operate under the current algorithm and, given the proper configuration, the swarm can complete missions of different requirements. For example, for search and rescue missions, the swarm can be set to operate under the advanced model which requires more time, but it is more efficient in terms of locating points of interest and collecting data from them. The swarm’s speed can also be tweaked to account for time-critical missions where any information collected in a short time frame can be useful.

The sections are organized as follows. In Section 2 an overview of related work is included. Section 3 details relative to the developed algorithm are provided. In Section 4 the simulated experiments are detailed, and an analysis is conducted on the results. Finally, in Section 5 conclusions are derived, and future work is provided.

## 2. Past Related Work

Many studies over the years have focused on drone swarm autonomy [17] and coordination using base stations [18]. The typical model of operation and coordination of drones remotely by a human pilot is slowly becoming part of the past. The move towards autonomy is the natural next step since digital systems can provide higher levels of accuracy as drones can be equipped with sensors [19] which provide increased navigation precision in natural environments. Additionally, drones can be tethered to nearby smart devices [20] which can provide location information effectively aiding towards the proper localization of drone swarms. This localization scheme can help a swarm conserve energy resources since this process does not require communication with satellites rather small-range, low-energy transmissions which can reach nearby devices.

Point-of-interest detection during the area coverage is an important goal of many studies which employ various technologies such as image-based deep learning and computer vision techniques [21,22]. Nature-inspired algorithms have been shown to provide very accurate results. A study published in 2018 [23] suggests an algorithm that can be adopted by a swarm of drones that use various environment-sensing techniques such as cameras or wireless signal detection.

Many applications include area monitoring using WSN-based systems [20,24,25]. Wireless sensory devices can be deployed on a wide area and relay sensed information back to a base station. Although this solution can provide accurate results, maintenance cost and possible failure can be deterring factors. In addition, typical WSNs are comprised of battery-powered nodes since the construction of a wired infrastructure requires large investments [26].

Wireless Sensor Networks are much easier to deploy. Studies have shown that by combining multiple algorithms such as the Particle Swarm Optimization (PSO) and Voronoi diagram [27] efficient WSN deployment can be achieved. Yet, WSNs operate using batteries, and wireless networking algorithms severely affected their active period since large amounts of information must be exchanged from specific nodes for the network to remain operational. This is known as the energy hole problem [28]. Drone swarms can potentially eliminate the issues that arise from statically deployed WSNs since they provide mobility, hence the system can be modified much easier.

Various Unmanned Aerial Vehicle (UAV) -based systems exist, all ranging in terms of communication capabilities and sensory devices included on the drones. This is attributed to the fact that different applications have unique requirements depending on which piece of environmental information is considered important. For instance, early fire detection and forest monitoring using drone swarms require cameras equipped with infrared and visible light sensors [16,29], while a model for area monitoring using drone swarms as mobile sink nodes which collect information from an installed WSN [30] might have requirements for different wireless antennas.

Drone swarm architectures can also include drones with various characteristics within the swarm [31] that can be used for crisis response applications such as major earthquake events. The architecture can include drones that operate at different altitudes and can serve a unique purpose. For instance, lower altitude Vertical Take-Off and Landing (VTOL) drones can collect information on sites where buildings have collapsed by employing deep penetrating radars or infrared cameras to locate trapped people. Drones of higher altitudes can provide real-time mapping of the area to assess the damage that has been inflicted on the area. And blimps—high-altitude balloons—due to their size can serve as the communications backbone for real-time collection of data from the swarm.

Decentralized algorithms can provide the necessary steps towards perimeter surveillance using a swarm of drones [32]. The drones are required to operate with information relative to nearby members of the swarm. No coordination is provided from outside the swarm. The drones have a single goal, monitor the entire perimeter using simple behavior by communicating with nearby drones. The drones occasionally require refueling since their flight time is limited, hence the algorithm provides dynamic behavior for drone removal and re-entrance in the area.

Applications that include area coverage where points of interest are known before the mission begins, can be categorized as Traveling Salesman Problem (TSP) for single pathfinding or Vehicular Routing Problem (VRP) where multiple routes are required [33,34]. Generally, combined behavior from multiple drones or a swarm of drones is referred to as Swarm Intelligence. This can include the path scheduling for all drones as well as drone response to unexpected events such as handling an obstacle and path rescheduling to avoid it.

Problems such as the TSP and VRP are NP-Complete optimization problems. Algorithms for team-based goal completion can also be found in nature. Bee colonies use simple processes to achieve their main goal which is honey production [35]. Bee Colony Optimization (BCO) has shown great potential towards solving optimization problems [20,36,37]. Agents which represent bees, follow simple steps and interactions which provide small portions of the required solution and thus contribute to solving larger problems.

Ant intelligence [38,39] has been observed to provide successful results in ant colonies. More specifically, ants can cooperate very efficiently without requiring complex behavior and work as a team to build very intricate structures where they store their food. The ant intelligence algorithm has shown potential for problems such as TSP [40] and VRP [41]. By providing approximate solutions to such problems, drones swarm applications, where area coverage with known points of interest is required, can greatly benefit [42].

Area coverage in environments with very little knowledge cannot be achieved with the methods described above. Rather, approaches that define algorithmic processes that aim towards the full area coverage and collection of information are required [43]. This problem is also known as the Coverage Path Planning (CPP) problem and it can be separated into three categories based on whether the AoI is split into subareas, which are covered separately, or a grid is used and when a drone reached the center of a square this is considered covered. The third category is called no decomposition where drones follow a path that covers the entire area without separating it. In the first case, the method is referred to as approximate cellular decomposition [44] while in the second, exact cellular decomposition [45].

Drones with miniature sizes can form a swarm which is ideal for indoor environment exploration. A study published in 2019 by McGuire et al. [46] describes an algorithm that can be employed for indoor exploration. It is based on area coverage concepts. The drones that form the swarm fly in multiple directions to cover the area more efficiently. Their starting point is a small base station which is used to attract the drones back when required. This is achieved using radio transmissions. When the drones receive the signal, they locate the point it originated from and fly towards that direction. The navigation in the area is achieved by tracking and following the walls.

The coverage path followed by the drones is an important aspect of area coverage and information collection. Studies have shown the effects of more rounded shapes such as the spiral and shapes with multiple edges [47]. This 2020 study has proven that coverage shape is closely related to the shape of the examined area. Areas with more edges, which tend to the circle, are being covered more efficiently from rounded coverage shapes, while shapes with fewer edges that tend to the square are better covered from parallel lines. Applications that require data collection from WSN have increased efficiency using circular coverage shapes [48].

Some studies focus on PoI detection during the area coverage, by employing various technologies such as image-based deep learning and computer vision techniques [21]. Nature-inspired algorithms have been shown to provide very accurate results. The authors in [23] propose an algorithm that can be adopted by a swarm of drones that use various environment-sensing techniques such as cameras or wireless signal detection. Their algorithm employs three nature-inspired processes, such as stigmergy, flocking, and evolution.

The coverage path followed by the drones is a key aspect of area coverage and information collection. The authors in [47] prove that the coverage path is affected by the area’s shape. Areas with more edges, which tend to the circle, are being covered more efficiently from rounded coverage shapes, while shapes with fewer edges that tend to the square are better covered from parallel lines. Applications that require data collection from Wireless Sensor Networks have increased efficiency using circular coverage shapes [48].

The literature review did reveal interesting algorithms for CPP, but full comparison is prohibited since our work does not share similarities during the simulation analysis. For example, in [49] the authors examine the coverage of public spaces for disinfection purposes using areal systems. Although they do analyze the area coverage using the parallel coverage path they only use a single drone to complete the coverage and there is no comparison with other coverage paths in terms of simulation time. To the best of the authors’ knowledge the analysis of the current work is original, and no other studies examine the current aspects of CPP in terms of PoI detection and specific algorithm behaviors. As such, no comparison with similar work is included in the sequel.

## 3. Autonomous Area Coverage Algorithm

Coverage of an area, hereafter referred to as the AoI, can be achieved using either single or multiple drones, and a distributed algorithm. The human-driven operation of a swarm is not efficient or effective enough since multiple drones can exist in it, thus, specifying the main behavior for autonomous operation can augment the swarm’s capabilities. The current section details the proposed algorithm that aims to tackle the full area coverage problem and the information collection from detected points of interest, to be referred to hereafter as PoI.

### 3.1. Swarm Formation

The drones consisting of the swarm are organized in a linear formation and their movement is in a straight line perpendicular to the formation. Each drone follows a path that is parallel to all the other paths in the swarm to prevent overlapping coverage paths.

The algorithm is designed with minimal requirements as initial information for each drone. The drones’ main goal is to obtain all required information during operating, including the one relative to the swarm and use it to achieve full area coverage based on the predefined behavior. The basic information describes a mission and includes the following: (i) Borders for the AoI with vertices $b_{i}$ that define a polygon; (ii) Movement direction $m_{d}=c$ for clockwise and *r* for counterclockwise; (iii) Area coverage path $c_{p}=p$ for parallel and *s* for spiral path planning, respectively; (iv) Coverage radius, $r_{c}$ measured in meters; and (v) Maximum navigation speed, $s_{n}$, used as the default speed which can be adjusted during missions based on current requirements.

It is assumed that a drone $u_{i}$ starts operating in a specific location without any prior knowledge of the swarm. First, it transmits a detection signal and joins a swarm with all detected drones. Then, all drones share their current location, $l_{0}$, their closest border vertex $b_{0}$ and the distance $d_{i}^{b}$ separating them from it, where *i* is the index of $u_{i}$. This process is depicted in Algorithm 1 as two function calls named receiveAllRemote() and addLocal().
**Algorithm 1:** Drone Swarm Border Align for Scanning.
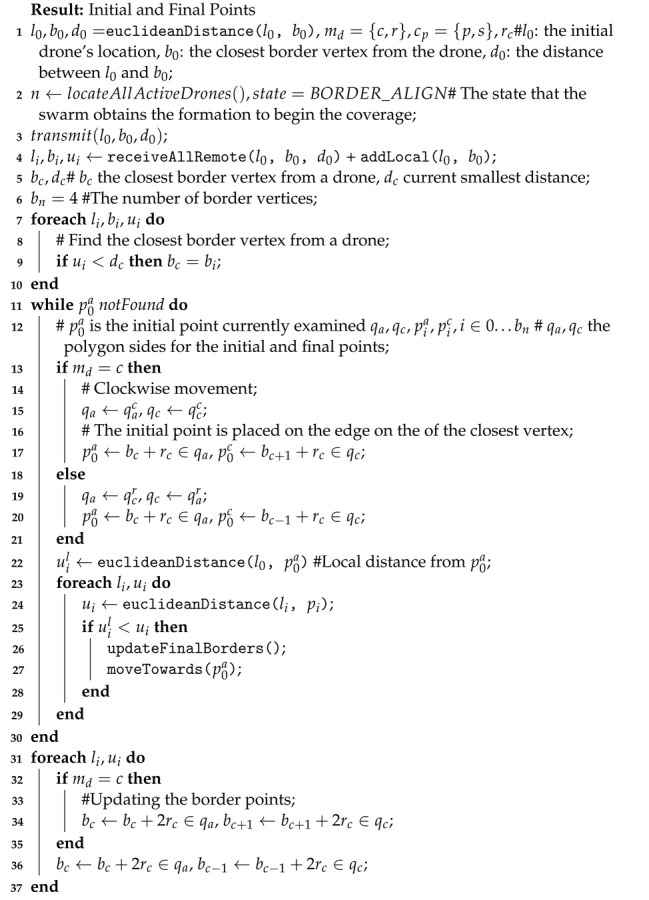


All drones now have collected the information related to the distances each one has from a vertex, and they can calculate the shortest distance. Then all drones move towards the one that has the closest area vertex, and obtain a linear formation. To achieve this, each drone calculates its initial point from which it starts covering the AoI. The Euclidean distance for two-dimensional planes is employed to calculate the distance between any two points in the area.

All initial points are located on a straight line that consists of the area’s borders and connects two of its vertices. The distance of two neighboring drones is equal to $2\times r_{c}$, where $r_{c}$ is the coverage radius, or the radius of the circular area a drone can cover each time instance. The final points for each partial linear coverage are also calculated. More details are provided in the sequel.

### 3.2. Partial Linear Area Coverage

Once the swarm obtains its formation and each drone reaches its location, they start covering the AoI. During the coverage process, they maintain equal distances from neighboring drones to avoid overlapping areas between them. The drones navigate from their initial to their final point in a straight line, something that helps maintaining their formation throughout the coverage process. Algorithm 2 details the process of the partial straight-line coverage. Once a part of the area is covered, it is removed from the AoI, hence a new area is assigned for the drones to cover that does not contain the already covered parts of the initial area. To re-enter the new AoI the swarm employs Algorithm 1. As a result, it alternates between the two algorithms until full area coverage is achieved.
**Algorithm 2:** Partial Area Coverage in Straight-Line Movement.
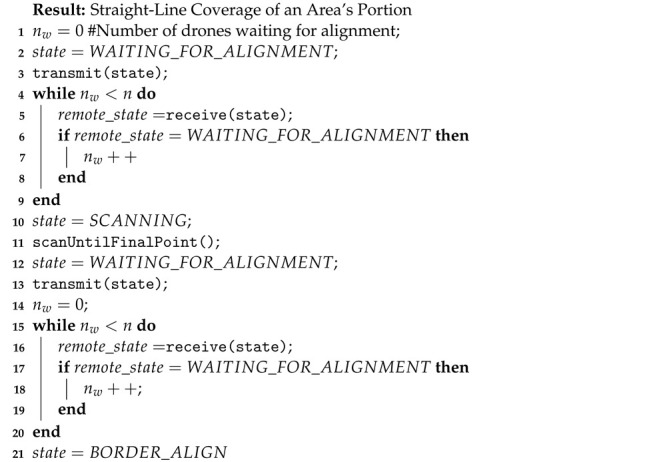


### 3.3. Point-of-Interest Detection Models

The drones have a specific coverage radius $r_{c}$ which once configured remains unchanged throughout a mission. When an amount of PoI is inside the $r_{c}$ the drone can choose one point at a time to collect information from. Two models are developed for this purpose, the basic and the advanced.

The drone estimates the time required for a PoI based on the amount of information needed from it and the time that it remains within its $r_{c}$. In the case where time is not enough, it reduces its speed and inform the swarm to adjust accordingly.

**Figure 1:**
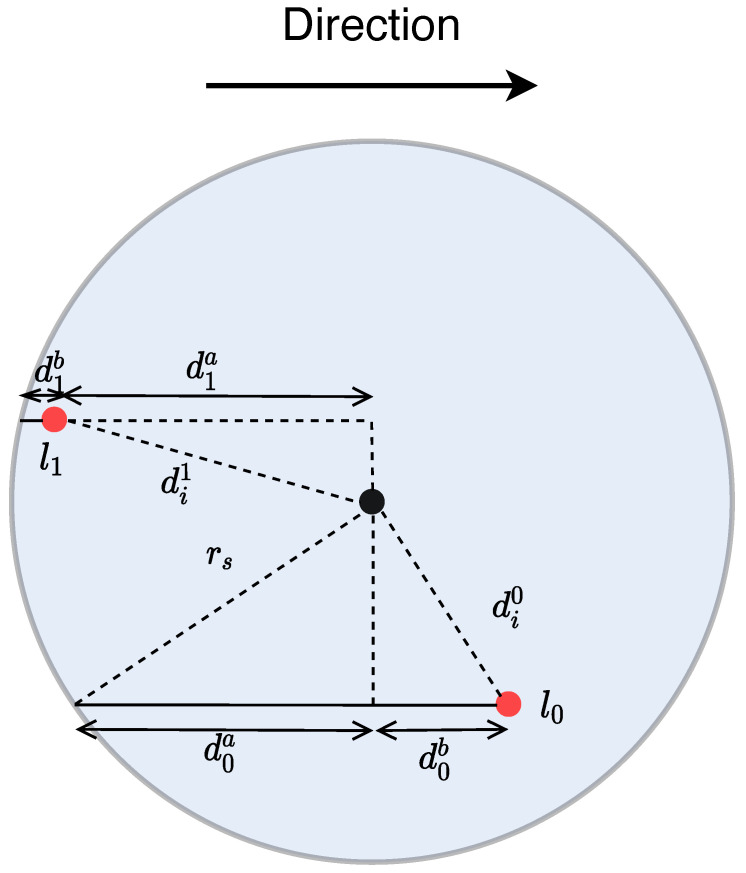
A drone with coverage radius $r_{c}$, represented by the black dot in the middle, discovering two points of interest PoI in different locations. The drone using the basic model chooses $l_{0}$ since it is closer compared to $l_{1}$. Contrary to that, using the advanced model, it chooses $l_{1}$ since it remains within its $r_{c}$ for less time.

Figure 1 depicts a drone which has detected two points $l_{0}$ and $l_{1}$. When the drone detects the points, their location in the map is acquired and, based on the drone’s current location, the distances $d_{i}^{0}$ and $d_{i}^{1}$ are calculated. In the basic model, $l_{0}$ is chosen first due to being closest to the drone and, as a result, $l_{1}$ is lost since it has exited the drone’s $r_{c}$ when information collection from $l_{0}$ is finished.

The advanced model improves the drone’s choice of PoI in a way that less PoIs are lost during the coverage. When it detects multiple PoIs the drone chooses the one that remains within its $r_{c}$ for less time. In the example of Figure 1 the drone’s first choice is $l_{1}$ since it remains within its $r_{c}$ for less time compared to $l_{1}$ thus, the drone has increased probability of not losing any PoI in the particular situation.

Although the swarm covers the AoI, the drones discover PoIs and collect information from them. It is assumed that a single drone can collect information from one PoI at a time. The drone estimates the amount of time required to completely collect the information based on the distance for which the PoI remains within the coverage radius. In the case where a PoI remains inside for less time than required, the drone reduces its speed and informs the swarm which also reduces its speed. When a PoI enters the coverage radius at its most remote point, the drone might have to drastically reduce its speed.

### 3.4. Coverage Paths

**Figure 2:**
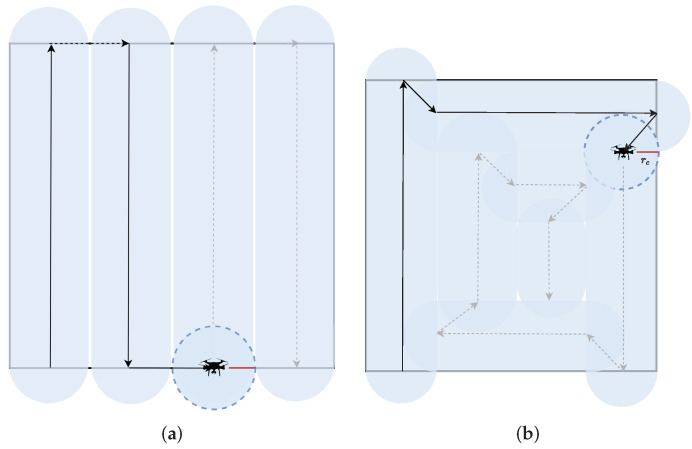
Area coverage paths of a single drone: (**a**) The parallel lines coverage path. The drone alternates its movement direction between clockwise and counterclockwise to achieve the parallel coverage path. (**b**) The spiral coverage path. The drone maintains a clockwise direction to achieve a spiral path for the area coverage.

The drones use a partial linear coverage which helps split the AoI and cover it partially until the full coverage. When the final point of each linear coverage is reached if the swarm maintains its $m_{d}$ then the final coverage path is a spiral. Otherwise, if it alternates between clockwise and counterclockwise the coverage path is parallel lines. Figure 2 depicts the parallel lines and spiral area coverage paths. The red line connecting the center of the drone with the edges of the coverage circle represents its $r_{c}$.

## 4. Simulation Results

To evaluate the algorithm’s effectiveness and efficiency, multiple simulation scenarios are considered under the OMNeT++ simulator. The main goal is to evaluate the algorithm’s ability to collect information from multiple PoIs within an AoI by employing a given number of drones in a swarm.

### 4.1. Simulated Environment and Parameter Setup

A swarm is considered within a simulated environment in which the parameters that determine its behavior are altered to evaluate the algorithm’s effectiveness. Different swarm sizes and multiple parameters are considered to alter the swarm’s capabilities among the simulation scenarios. The simulations conclude when the swarm has successfully covered the AoI and the parameters are retained at this point. The swarm sizes are set to n=1,2,4. The drone’s coverage radius is $r_{c}=25,50,100$ m. The amount of PoI ranges between 25 and 300 and is incremented by 25 between simulated experiments. To produce more accurate results the simulations are executed 20 times with variable PoI locations in the AoI.

The parameters that are retained for the analysis are the swarm’s mean speed $\bar{s}$ measured in meters per second (m/s), the time *t* required for the coverage of the area measured in seconds (s), the percentage of PoI detected depicted as $z_{j}\left(\%\right)$, the number of slowdowns *w* executed by the swarm, the mean travel distance $d_{i}$ per drone in meters (m) and the mean number of switched formations $\bar{f}$ until the full area coverage. Table 1 depicts all parameters used for the current simulations.

### 4.2. Results

In the sequel, a distance analysis is conducted to evaluate the performance of the algorithm in terms of full area coverage, and more specifically the total distance traveled by the swarm. In addition, a general performance analysis takes place to determine algorithm’s effectiveness in collecting data from newly discovered PoIs using variable swarm sizes and coverage paths. To properly demonstrate the simulation results, every experiment is executed 20 times thus, acquiring mean values for all metrics is a prerequisite for the final visualization of the swarm’s behavior.

#### 4.2.1. Full Coverage Distance Analysis

**Figure 3:**
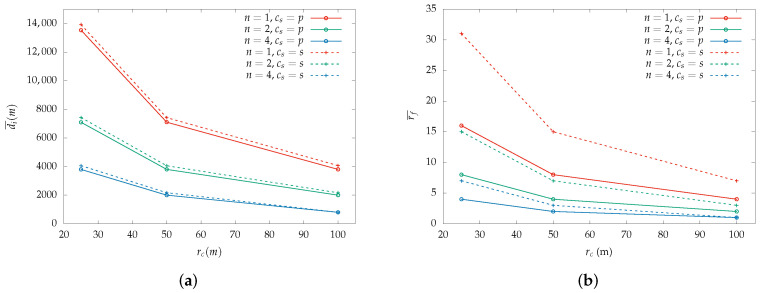
Mean distance a drone traveled during the simulations for a swarm size n=1,2,4 based on the coverage radius $r_{c}$: (**a**) Mean distance per drone $\bar{d_{i}}$ while the $r_{c}$ is increased. (**b**) Mean number of switched formations $\bar{f}$ for parallel and spiral coverage paths based on variable $r_{c}$. The dashed lines represent the spiral coverage path while the parallel is depicted with a solid line. The results are derived using the basic PoI detection model. Identical results are depicted using the advanced model since a similar AoI is used for both.

Figure 3 depicts the total distance traveled by a drone with coverage radius $r_{c}=25,50,100$ m. The comparison between the two paths showcases that the spiral requires more travel distance compared to the parallel path which are depicted with dashed and solid lines, respectively. In both paths, when the number of drones is reduced by half, the travel distance for the same $r_{c}$ is doubled, which is quite reasonable since the size of the area that requires coverage remains the same.

The area coverage path affects the required travel distance for the complete area coverage. This is attributed to the fact that each path requires a different number of switched formations for the same area size. For example, the travel distance when the coverage radius is equal to 25 m for the parallel lines is 13,524 m while for the spiral is 13,918 m. A swarm must alternate between formation change and partial straight-line coverage more times for the spiral path and this leads to excess travel distance since for every formation switch, the drones must re-enter the AoI, hence for that time they fly outside the new borders.

Each time the swarm switches its formation, if it maintains its $m_{d}$—the spiral path—the next area side it aligns with is smaller. This behavior gradually reduces the area’s sides and thus, the swarm must switch its formation more times compared to the parallel path to completely cover the area. The results depict that the spiral path introduces an average of 5% increase in the required time for the same area size compared to the parallel path. The percentage of detected points of interest is hardly impacted between the two coverage paths.

The distance outside the borders $d_{o}$ can be estimated for both coverage paths. For the parallel lines, this distance is:

(1) $$d_{o}^{p}=2r_{c}fp_{a}^{c},$$

where $p_{a}^{c}$ is the number of initial points to check until the closest one is found, and *f* is the number of formation switches until the area is fully covered. The distance outside the borders for a drone covering an area with a spiral coverage path is:

(2) $$d_{o}^{s}=f\sqrt{{\left(d_{b}^{l}\right)}^{2}+{\left(2r_{c}\left(p_{a}^{c}-1\right)+r_{c}\right)}^{2}}.$$

The $d_{b}^{l}$ is the distance from the closest border point. The first initial point that is calculated has a distance of $r_{c}$ from the drone while the rest of the initial points have a distance $r_{c}\left(p_{a}^{c}-1\right)+r_{c}$. These distances constitute an orthogonal triangle, and its hypotenuse is $d_{o}$. There are multiple initial points for each formation switch only when n>1.

Based on the distance analysis, it is derived that the coverage time is also affected by the coverage path independently of the $r_{c}$ size. A comparison between the figures that depict the parallel and spiral coverage paths reveals that in all cases the spiral path demands extended flight time from the swarm. Thus, a coverage path that requires fewer formation switches can benefit the swarm in terms of flight required time.

The advanced and basic PoI detection models do not affect the travel distance required for the same area size. The measured mean speed per drone is decreased as more drones are added since the entire swarm adjusts its speed to retain the same formation. Though, time is reduced overall since more drones cover the same area. Additionally, more time is required for the advanced model to fully cover the AoI and, also, the mean speed per drone is lower compared to the basic model.

#### 4.2.2. General Performance Analysis

**Figure 4:**
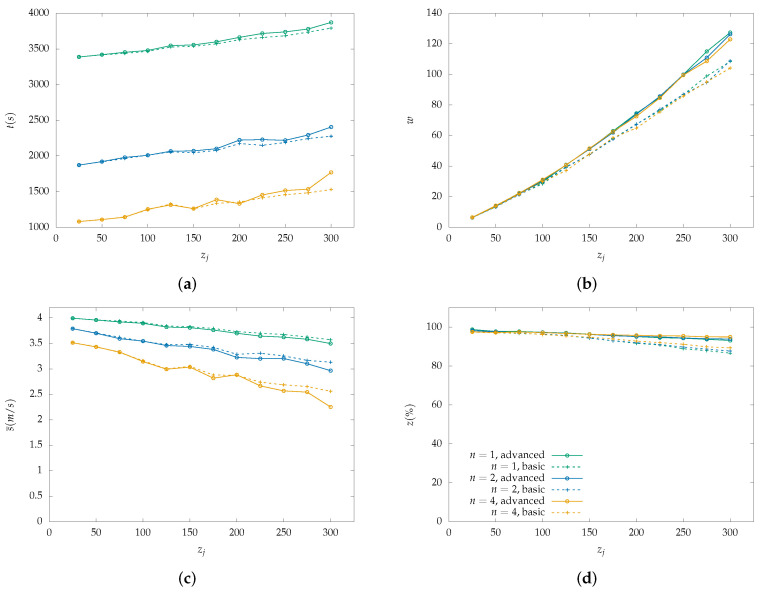
Simulation results for a swarm with 1, 2 and 4 drones with $r_{c}=25$ m and parallel lines coverage path. Two PoI detection models are depicted here, the basic with dashed lines and the advanced with solid lines: (**a**) Cover time *t* in seconds by the initial number of PoI. (**b**) Number of slowdowns *w* required for the information collection from the PoI. Depends on the number of PoI that exist inside the map. (**c**) Mean speed $\bar{s}$ measured in meters per second (m/s) throughout the simulation with a variable number of initial PoI. Depends on the number of slowdowns. (**d**) Percentage of scanned PoI while their number increases, depicted as z(%). (Green color): Single drone swarm; (Blue color): Two-drone swarm; (Orange color): Four-drone swarm.

**Figure 5:**
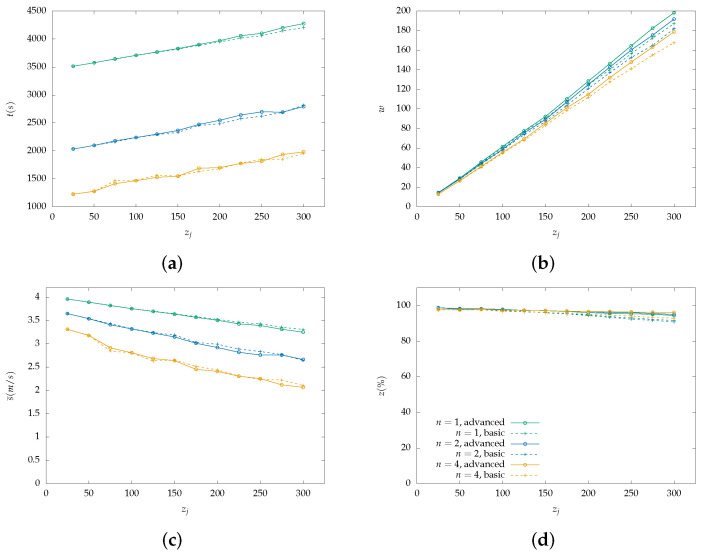
Simulation results for a swarm with 1, 2 and 4 drones with $r_{c}=25$ m and spiral lines coverage path. Two PoI detection models are depicted here, the basic with dashed lines and the advanced with solid lines: (**a**) Cover time *t* in seconds by the initial number of PoI. (**b**) Number of slowdowns *w* required for the information collection from the PoI. Depends on the number of PoI that exist inside the map. (**c**) Mean speed $\bar{s}$ measured in meters per second (m/s) throughout the simulation with a variable number of initial PoI. Depends on the number of slowdowns. (**d**) Percentage of scanned PoI while their number increases, depicted as z(%). (Green color): Single drone swarm; (Blue color): Two-drone swarm; (Orange color): Four-drone swarm.

**Figure 6:**
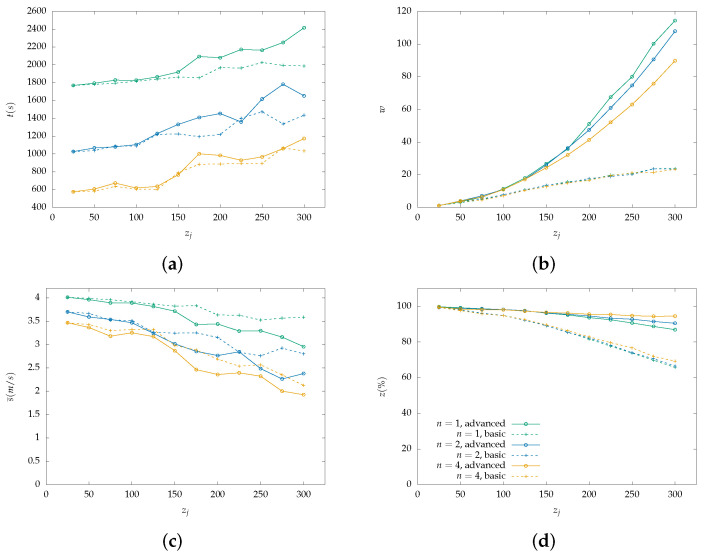
Simulation results for a swarm with 1, 2 and 4 drones with $r_{c}=50$ m and parallel lines coverage path. Two PoI detection models are depicted here, the basic with dashed lines and the advanced with solid lines: (**a**) Cover time *t* in seconds by the initial number of PoI. (**b**) Number of slowdowns *w* required for the information collection from the PoI. Depends on the number of PoI that exist inside the map. (**c**) Mean speed $\bar{s}$ measured in meters per second (m/s) throughout the simulation with a variable number of initial PoI. Depends on the number of slowdowns. (**d**) Percentage of scanned PoI while their number increases, depicted as z(%). (Green color): Single drone swarm; (Blue color): Two-drone swarm; (Orange color): Four-drone swarm.

**Figure 7:**
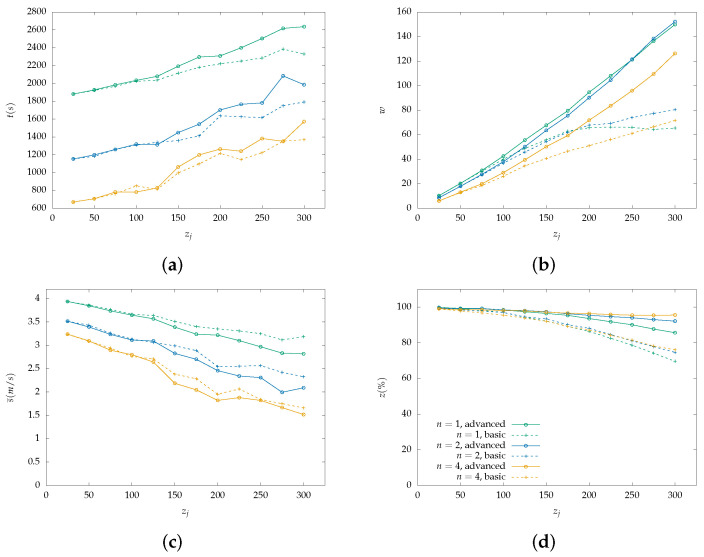
Simulation results for a swarm with 1, 2 and 4 drones with $r_{c}=50$ m and spiral lines coverage path. Two PoI detection models are depicted here, the basic with dashed lines and the advanced with solid lines: (**a**) Cover time *t* in seconds by the initial number of PoI. (**b**) Number of slowdowns *w* required for the information collection from the PoI. Depends on the number of PoI that exist inside the map. (**c**) Mean speed $\bar{s}$ measured in meters per second (m/s) throughout the simulation with a variable number of initial PoI. Depends on the number of slowdowns. (**d**) Percentage of scanned PoI while their number increases, depicted as z(%). (Green color): Single drone swarm; (Blue color): Two-drone swarm; (Orange color): Four-drone swarm.

**Figure 8:**
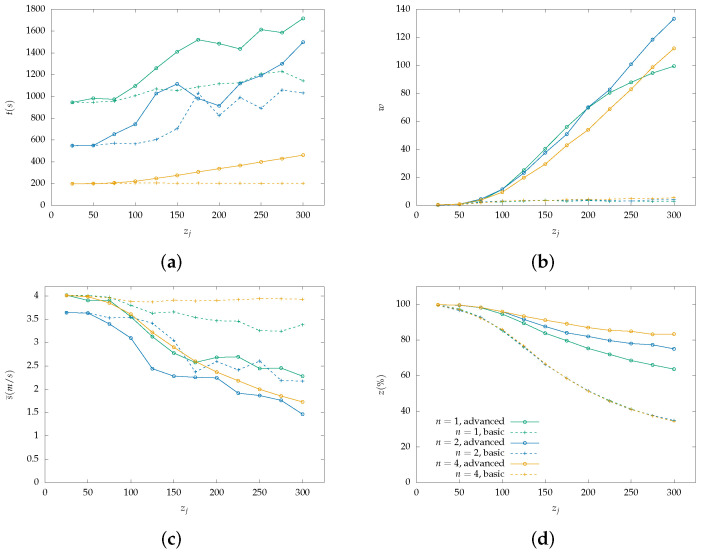
Simulation results for a swarm with 1, 2 and 4 drones with $r_{c}=100$ m and parallel lines coverage path. Two PoI detection models are depicted here, the basic with dashed lines and the advanced with solid lines: (**a**) Cover time *t* in seconds by the initial number of PoI. (**b**) Number of slowdowns *w* required for the information collection from the PoI. Depends on the number of PoI that exist inside the map. (**c**) Mean speed $\bar{s}$ measured in meters per second (m/s) throughout the simulation with a variable number of initial PoI. Depends on the number of slowdowns. (**d**) Percentage of scanned PoI while their number increases, depicted as z(%). (Green color): Single drone swarm; (Blue color): Two-drone swarm; (Orange color): Four-drone swarm.

**Figure 9:**
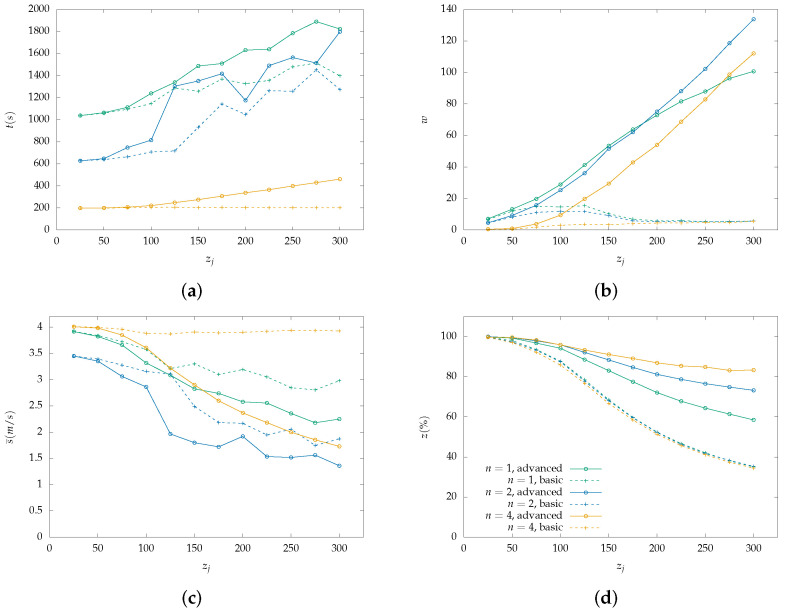
Simulation results for a swarm with 1, 2 and 4 drones with $r_{c}=100$ m and spiral coverage path. Two PoI detection models are depicted here, the basic with dashed lines and the advanced with solid lines: (**a**) Cover time *t* in seconds by the initial number of PoI. (**b**) Number of slowdowns *w* required for the information collection from the PoI. Depends on the number of PoI that exist inside the map. (**c**) Mean speed $\bar{s}$ measured in meters per second (m/s) throughout the simulation with a variable number of initial PoI. Depends on the number of slowdowns. (**d**) Percentage of scanned PoI while their number increases, depicted as z(%). (Green color): Single drone swarm; (Blue color): Two-drone swarm; (Orange color): Four-drone swarm.

Each figure from Figure 4, Figure 5, Figure 6, Figure 7, Figure 8 and Figure 9 is a set of four figures with a common x-axis value, the amount of PoI in the AoI. More specifically, figure (a) depicts the time required to completely cover the area, figure (b) depicts the number of times the swarm must reduce its speed to collect information, figure (c) depicts the mean speed per drone during the simulation, and figure (d) depicts the percentage of PoI from which information is collected.

Two models of PoI detection are examined, the basic and the advanced which are represented with a dashed line and solid one, respectively. Figure 4, Figure 6 and Figure 8 depict the results for the parallel coverage path while Figure 5, Figure 7 and Figure 9 depict the ones for spiral coverage path for $r_{c}=25,50,100$ in both cases, respectively.

The advanced PoI detection model provides a more sophisticated selection process compared to the basic one. Thus, the swarm can collect information from more PoI in the AoI. This is observed for all $r_{c}$ values and both parallel and spiral coverage paths. Though, for larger $r_{c}$ values the benefits are greater. Small $r_{c}$ values benefit the swarm since each drone must calculate which PoI to select from a smaller area and thus, from a smaller collection of PoIs. The swarm’s moving speed is between 0.5 m/s and 4 m/s and since a drone collects information from a single PoI at a time smaller $r_{c}$ values help it focus more on the area it is covering.

Increasing the swarm size has two effects on the overall performance. First, the required time for full coverage is reduced, and second, the percentage of PoIs that it can detect is increased. To better understand the second effect, it is important to consider the algorithm functionality on PoI detection. For a swarm with n=2 and $r_{c}=100$ m drone $u_{1}$ requests a speed reduction and drone $u_{2}$ must follow to remain in the same formation. Despite $u_{2}$ not requiring the extra time, it benefits from the speed reduction since it remains in the same area for a longer time which helps it collect information from more PoI before they leave its radius. A point $z_{1}$ can be ignored when a drone is collecting information from another $z_{0}$ while at a certain speed which results in $z_{1}$ exiting the drone’s coverage radius.

In the case where $r_{c}=100$ and n=4 both parallel and spiral coverage paths provide similar results since the area has a size of 800×800 and $2\times r_{c}\times n=800$ thus, the area is covered in both cases without any formation changes. It is important to note that while the number of PoIs remains low, both the basic and advanced models have similar behavior and can detect almost all PoIs but when their number is increased above 25, the advanced model has an obvious advantage. Additionally, in the first case, all metrics depicted in Figure 7 and Figure 8 have similar behavior to the one described above.

## 5. Conclusions and Future Work

In conclusion, path planning for a defined AoI can be achieved with multiple autonomous drones organized as a swarm. Point-of-interest detection is affected by the coverage path followed by the drones. Based on the simulated results, a path is considered more efficient when the drones do not need to switch their formation an excessive number of times to fully cover the area. Two coverage paths are examined in the current work, parallel and spiral. The latter requires more formation switches compared to the former thus, it introduces an extra 5% time increase for the same area size. Two models for PoI detection are proposed, the basic where the closest point is selected and the advanced which calculates the amount of time all PoIs remain within the coverage radius and selects the one that leaves it first. The advanced model introduces a small increase in the required time for the coverage of the area but while the number of points of interest is increased, it helps the swarm detect more of them compared to the basic model.

Future work will include further simulations which will help determine how the PoI distribution in the area affects the swarm’s ability to collect information from them. Additionally, an analysis will be introduced for the effects of different movement directions for the same distributions of points of interest. An extension to the drones will be applied to allow for data collection from multiple PoIs at the same time with variable connection and disconnection times. Additionally, the algorithm will be improved to allow the drones to ask for assistance from neighboring ones which might will limit data loss from PoIs. Additionally, an extensive analysis relative to the computational and time complexity will be included as well as real-world application will be implemented to provide insight for the algorithm’s effectiveness in more complex environments. Finally, simulations with an increased number of drones will be conducted and more complex area shapes will be examined.

## Figures and Tables

**Table 1:** Main simulation parameters.

Name	Symbol	Potential Values
Swarm size	*n*	1, 2, 4
Coverage radius	$r_{c}$	25, 50, 100
# of PoIs	$z_{j}$	25, 50, 75, …, 300
Movement direction	$m_{d}$	*c*, *r*
Swarm maximum speed	*s*	4 m/s or 14.2 km/h
Swarm mean speed	$\bar{s}$	-
Mean travel distance per drone	$d_{i}$	-
Distance outside borders	$d_{0}$	-
# of formation switches	*f*	-
Time for coverage	*t*	-
# of slowdowns	*w*	-
Mean # of formation switches	$\bar{r_{f}}$	-

## Data Availability

Not applicable.
